# Retroperitoneal Pseudoaneurysm Mimicking Ureteral Calculus: Pitfalls in Diagnosis

**DOI:** 10.7759/cureus.1758

**Published:** 2017-10-08

**Authors:** Aleksandr Kalabin, Vishnu R Mani, Anant Dinesh, Marina Landa, Brian Davis-Joseph

**Affiliations:** 1 Department of General Surgery, Columbia University College of Physicians and Surgeons at Harlem Hospital Center; 2 Department of Surgery, New York University School of Medicine, and the Laura and Isaac Perlmutter Cancer Center, Columbia University School of Physicians and Surgeons at Harlem Hospital Center; 3 Pathology, Columbia University College of Physicians and Surgeons at Harlem Hospital Center; 4 General Surgery and Urology, Columbia University College of Physicians and Surgeons at Harlem Hospital Center

**Keywords:** pseudoaneurysm, ureteral calculus, misdiagnosis, hematoma

## Abstract

Arterial aneurysms (AA) can be classified as true aneurysms, characterized by the persistence of all three layers of the arterial wall with progressive dilation and wall thinning; arterial pseudoaneurysms (APAs) are characterized by a tear in the vessel wall and a periarterial hematoma formation. They could occur due to a visceral, retroperitoneal, or peripheral origin. Most AA/APA are usually found incidentally, and it is imperative to be vigilant in order to diagnose and manage them due to their potentially life-threatening complications. We present a case of a 35-year-old woman presenting with right-sided abdominal pain mimicking renal colic with an initial misdiagnosis of ureteral calculus. Post-cystoscopy, a misdiagnosis was confirmed, and subsequently, the patient had a right retroperitoneal mass excision. The histopathology report concluded the calcified retroperitoneal mass to be pseudoaneurysm. Such pitfalls in diagnosis are essential to be shared with the larger medical community for increased vigilance and optimal management of pseudoaneurysms.

## Introduction

There are no known reported data on the incidence of retroperitoneal arterial aneurysms and arterial pseudoaneurysms (AA/APA). However, visceral artery aneurysms (VAA) and visceral artery pseudoaneurysms (VAPA) are relatively rare, with a reported incidence of 0.01 to 0.2% in routine autopsies [1]. The most common site of occurrence of VAA and VAPA are splenic, hepatic, gastroduodenal, pancreaticoduodenal, superior mesenteric, and inferior mesenteric arteries. A true aneurysm is defined by its intact tri-layer vessel wall structure, e.g., intima, media, and adventitia, with progressive dilatation and thinning, whereas in APA, there is a breach in all three vessel wall layers, usually from a tear secondary to trauma with subsequent periarterial hematoma formation. As retroperitoneal aneurysmal data has not been accounted for in the literature, it is important to mention that the rate of life-threatening complications is much higher in VAPA compared to VAA. For instance, the rate of VAPA rupture is 76.3% compared to only 3.1% for VAA. At the same time, aneurysm size is not a reliable predictor of rupture [2]. VAPAs are usually asymptomatic and diagnosed incidentally. When symptomatic, they could present with symptoms based on the location of VAPA and its ensuing potential complications (as described below in the discussion). Our case, as described in this report, reinforces the need for all medical practitioners to have a broad differential diagnosis and to especially include the atypical presentation of life-threatening conditions, such as VAPAs and retroperitoneal aneurysms.

## Case presentation

A 35-year-old woman with no past medical and surgical history presented to the emergency department with severe colicky right flank pain radiating to right lower quadrant. The patient reported that the pain started two weeks previously and had been worsening since the last few days.

**Figure 1:**
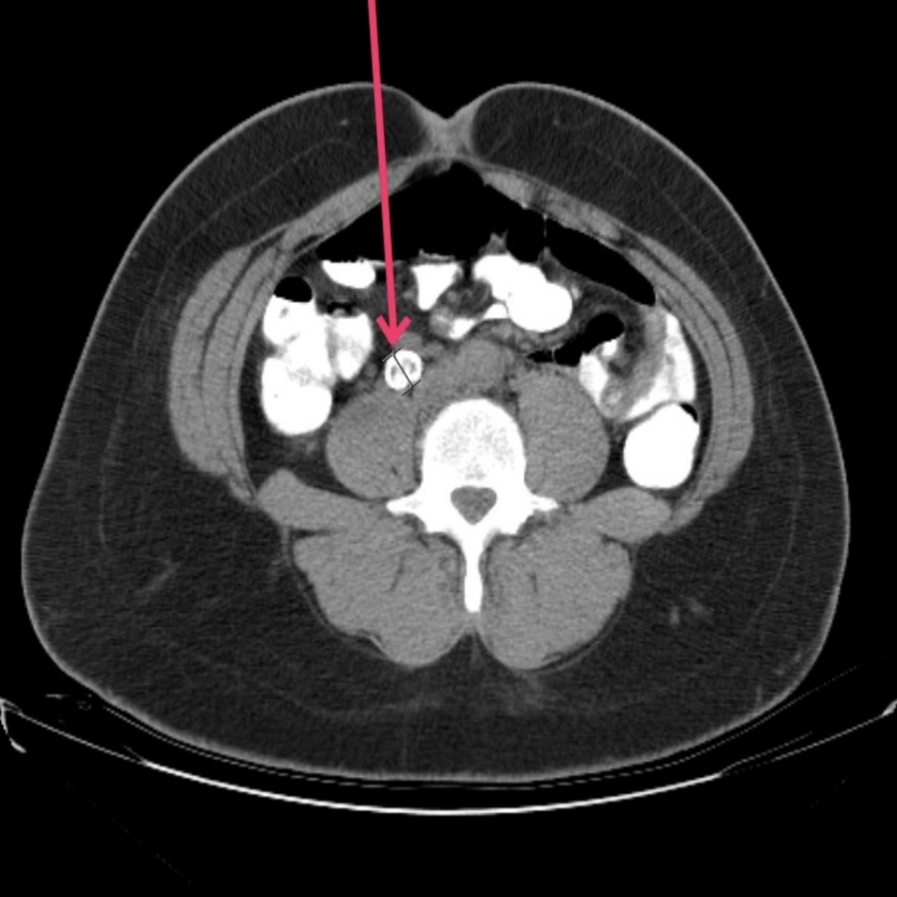
Computed tomography of the abdomen and pelvis Right retroperitoneal high density mass (red arrow) on the anterior surface of psoas muscle.

A complete history was taken and physical exam performed, along with comprehensive laboratory and radiological evaluation. Elevated white blood cell (WBC) count with borderline renal function was noted, and an abdominal computed tomography (CT) was done without intravenous contrast (Figure 1). This demonstrated a calcified 1.5 cm mass next to anterior aspect of right psoas muscle, which was misdiagnosed as possible ureteral calculus, and a presumed diagnosis of obstructive uropathy was established.

After the diagnosis was made, the patient was admitted to the urology service, actively resuscitated with fluids, given pain medication, and observed. She deteriorated during the course of admission with increasing fever, chills, and worsening pain. We obtained repeat blood work along with the cultures from blood and urine, and the patient was immediately taken to the operating room for cystoscopy, retrograde ureterography, and ureteral stenting as a temporary measure of managing obstructive uropathy. The patient was taken to the operating room where cystoscopy revealed orthotopic ureteral orifices with unremarkable bladder architecture. An intraoperative stent was placed, and fluoroscopic examination revealed the presence of extra-ureteral lesion with calcification and the absence of any filling defect. The ureter ran medial to the calcification, and the J stent was left in situ. She was informed of her findings and was advised to have further investigation and management by excisional biopsy. However, the patient left the hospital against medical advice.

**Figure 2:**
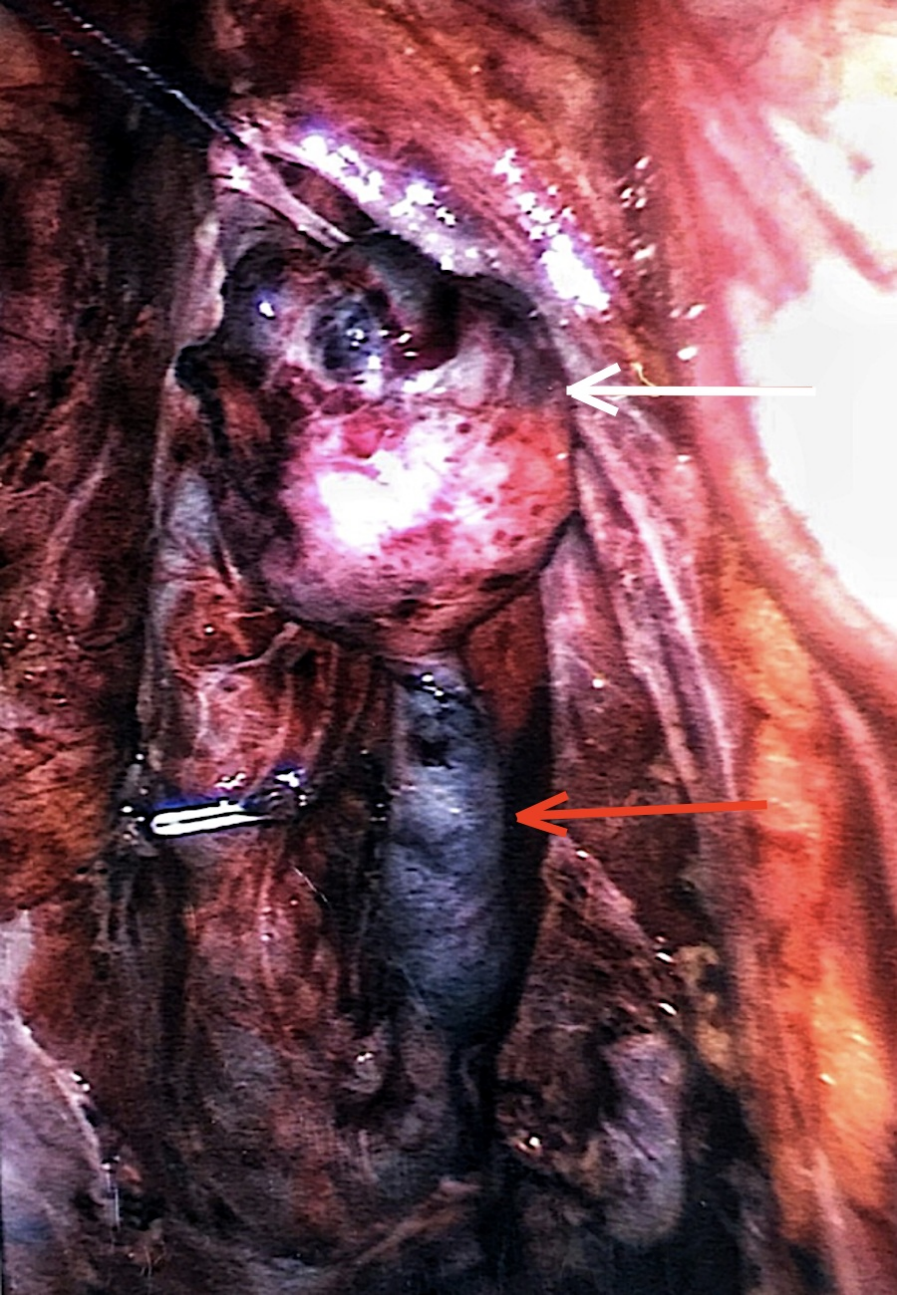
Right retroperitoneal mass Intraoperative finding of right retroperitoneal firm mass (white arrow), measuring 1.5 cm in greatest dimension with attached rubbery tail-like tissue (red arrow)

She returned to the emergency room after three months, complaining of persisting right flank pain that was on and off, and was subsequently re-admitted to the urology service. Repeat CT scan confirmed the persistence of the mass. She consented for diagnostic laparoscopy and possible excisional biopsy of the mass. She was subsequently taken to the operating room where a laparoscopic evaluation was done, and the right retroperitoneal mass was noted (Figure 2), which was subsequently resected without any complications or difficulties. All intra-abdominal structures were evaluated and were unremarkable.

The patient was transferred to the post-anesthesia care unit (PACU) and was given optimal postoperative care.

**Figure 3:**
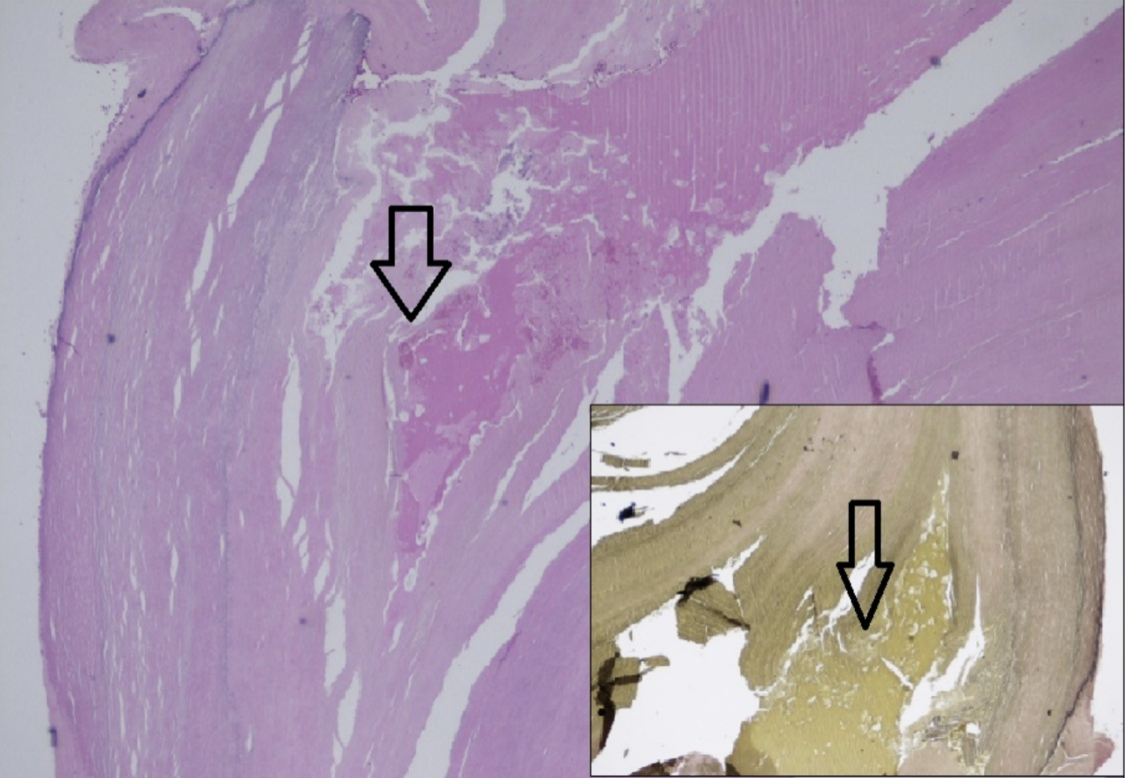
Final pathology report Pathology slide (H & E stain) shows cross-section of arterial segment exhibiting a breach in the vessel wall. The vascular wall is extensively collagenized and partially calcified. Inset: Van Gieson stain highlights the breach in the vessel wall.

The specimen was dissected, and on removal, we noted an ellipsoid mass with a thick capsule which was beige in color, with a septae compartmentalizing a darker center. The proximal end had a indurated pellet of uncertain composition. We sent it for further histopathological exam which reported right retroperitoneal mass with changes consistent with pseudoaneurysm wall (Figure 3). The specimen was extensively collagenized and partially calcified without any evidence of ectopic organs or neoplasia.

## Discussion

Pseudoaneurysm, false aneurysm, pulsatile hematoma, and communicating hematoma are synonymous terms and may be used interchangeably. Pathologically, the wall of the arterial pseudoaneurysm has been breached; the external wall of the aneurysmal sac consists of arterial layers with perivascular tissue, blood clot and/or layers of reactive fibrosis and might undergo further changes with time, like calcification [3]. Retroperitoneal and visceral artery pseudoaneurysms most commonly occur as a result of trauma, infection, atherosclerosis, inflammation, neoplasm, iatrogenic injury from endovascular interventional radiological or surgical procedures, vasculitis, and extension of aortic dissection [4].

VAA, VAPA, and retroperitoneal aneurysms most commonly involve celiac trunk, the superior, inferior mesenteric, renal arteries, and their branches. The pathogenesis of pseudoaneurysm is poorly established and may be associated with aforementioned risk factors, but sometimes might be idiopathic as in our case where the young female patient did not have the aforementioned associations. Clinical presentation of individual VAA/VAPAs differs depending on anatomic location, size, and organ associations. Though often asymptomatic [5], the incidence of rupture varies from 25% to 70% and is usually not related to aneurysmal size [6]. Untreated mortality rates could reach as high as 90% [4]. Owing to the disruption of all three layers of the arterial wall with a hematoma, they are at high risk of impending rupture. Therefore, treatment of retroperitoneal and visceral pseudoaneurysm is imperative without any delays, with surgery and radiological intervention as the first treatment of choice. Incidental detection of such vascular anomalies are feasible by advanced imaging modalities and increasing adoption of computed tomography and angiography for unrelated reasons. For instance, visceral angiography has almost 100% sensitivity as it helps delineate anatomy and allows for both diagnostic and therapeutic intervention [7].

Despite recent advances in surgical techniques and diagnostic tools, management of visceral artery aneurysms remains extremely challenging. There is no consensus on the optimal management of VAPA and retroperitoneal aneurysms (surgery v/s endovascular procedure) [8-9] due to the low incidence and absence of controlled studies. As APAs represent a contained rupture that is only constrained by a fibrous capsule and have a high risk for rupture compared with a true aneurysm as described above, the general approach could be summarized as early elective treatment rather than a "wait and watch" strategy in order to avoid life-threatening complications.

VAA/VAPA and retroperitoneal aneurysms commonly can be treated with endovascular intervention (covered stent and coiled embolization) or operative repair that consists of exclusion of the aneurysmal sac from the systemic circulation while ideally preserving distal blood flow. Current literature indicates that open and endovascular approaches have similar rates of technical and clinical success. However, it should be duly noted that the endovascular approach has resulted in rupture of pseudoaneurysm and bleeding which has been reported [10]. In case of either an acutely expanding pseudoaneurysm or the patient's clinical deterioration, an urgent open repair would be required. As described in detail, the importance of discussing VAPA and retroperitoneal aneurysms is associated with reports of atypical presentation in literature, such as the absence of characteristic symptoms or history of trauma/surgical intervention. It has also been reported to be mimicking a sarcoma and, in some cases, presenting with jaundice and pruritus of an unknown etiology. 

## Conclusions

From our search of literature, we were unable to delineate the exact or approximate distribution percentage of retroperitoneal or VAPAs and the frequency of their occurrence and hence, their rarity and importance of awareness and vigilance, although with an increasing number of reported cases, they might be present more and could often be missed during diagnosis. Our case is unique and multivariate, featuring a misdiagnosis of ureteral calculi, and later, an intraoperative diagnosis of a vascular mass. We confirmed the diagnosis to be a pseudoaneurysm. Our report clearly demonstrates the low threshold for prudence with regards to VAA, VAPA, and retroperitoneal aneurysms in our day-to-day differentials. Through this, we would like to emphasize the importance of vigilance in our day-to-day practice of this diagnosis.
